# Opposing effects of sugar-free claims on perceived healthiness and sweetness reduce consumers’ willingness to pay for sugar-free products

**DOI:** 10.3389/fnut.2025.1644753

**Published:** 2025-10-31

**Authors:** Ksenia Panidi, Yaroslava Grebenschikova, Vasily Klucharev, Anna N. Shestakova

**Affiliations:** ^1^Centre for Cognition and Decision Making, Institute for Cognitive Neuroscience, HSE University, Moscow, Russia; ^2^International Laboratory of Social Neurobiology, Institute for Cognitive Neuroscience, HSE University, Moscow, Russia

**Keywords:** sugar-free products, willingness to pay, Becker-deGroot-Marschak auction, sugar-free label, healthiness, tastiness

## Abstract

**Background:**

Overconsumption of sugar-enriched food remains one of the leading causes of obesity around the world. However, the question of whether consumers are willing to substitute sugar-containing products with their sugar-free analogues remains underexplored. One factor affecting consumers’ choices is their willingness to pay for sugar-free products. In the present study, we test the hypothesis that consumers are willing to pay more for sugar-free labeled products compared to their sugar-containing analogues, and that this effect is mediated by the subjective perceptions of product healthiness, tastiness, and sweetness induced by the label.

**Methods:**

In our experiment, participants placed bids for sugar-containing and analogous sugar-free products in a Becker-deGroot-Marschak auction to determine their willingness to pay. Additionally, they rated each product on the level of perceived healthiness, sweetness, tastiness, and familiarity with the product. We then used structural equation modeling to estimate the direct, indirect, and total effect of the label on the willingness to pay.

**Results:**

The results suggest that, controlling for familiarity with the product, sugar-free labels significantly increased the willingness to pay due to the perception of sugar-free products as healthier than sugar-containing ones. However, this positive effect was overridden by a significant decrease in perceived tastiness and sweetness of products labeled as sugar-free compared to sugar-containing ones, which in turn led to a reduction in the willingness to pay. The overall effect of the label on the willingness to pay was, thus, insignificant. Additionally, we show that the effect of the label on perceived tastiness was fully mediated by perceived sweetness.

**Conclusion:**

The opposing effects of the label on subjective product perceptions may be limiting the efficiency of sugar-free claims in changing consumer choices towards healthier food options.

## Introduction

According to a recent World Health Organization report, noncommunicable diseases, including cardiovascular diseases and diabetes, account for more than 70% of preventable premature deaths worldwide (1). Obesity is one of the causes of chronic disorders, owning to an overconsumption of ultra-processed foods high in sugar (2, 3). Substituting harmful food for healthier alternatives is not only a matter of these items’ organoleptic properties, but also a matter of price. Some studies have found that healthier food items may be more expensive than their less healthy counterparts. According to a study conducted in Northwestern Mexico, sugar-free, sugar-reduced, and low-glycemic-index cereal products have lower market availability and significantly higher pricing than their conventional analogues (4). High energy-density foods and less healthy beverages may cost less per serving and per 1,000 calories (5). Furthermore, prices for healthy and unhealthy products may rise disproportionately, with healthy product prices increasing more than those of unhealthy food items (6). Consumers may also hold the lay belief that healthier food items usually cost more than unhealthy ones even in cases when this is not necessarily true (7, 8). As a result, consumers’ dietary choices, particularly those with reduced sugar content, may be substantially influenced by their willingness to pay (WTP) for healthier food as opposed to less healthy analogues.

Furthermore, nutritional labels on food packaging, such as those notifying consumers about the increasing amount or lack of refined sugar, influence their dietary choices (9, 10). However, the debate over whether sugar-content labeling is effective in shifting consumers’ preferences towards healthier products is still ongoing. A recent systematic review revealed that sugar-related warning labels are effective in encouraging healthier choices for drinks with high sugar content (11). A recent study found that warning labels on high-sugar drinks significantly reduced the choice of sugar-sweetened beverages (12). The implementation of the government sugar labeling regulations reduced household purchases of soft drinks with high sugar contents in Chile by 23.7% (13). Although sugar labeling systems may be effective in some contexts, other studies found no significant change in sales following the introduction of a color labeling system with green labels for sugar-free and red for high sugar content (14) or found an increase in buying intentions for products with health-related properties only in a subgroup of participants (15). Interestingly, a meta-analysis of studies involving various food labeling systems indicated that labels had no significant effect of labels on total carbohydrate consumption, whereas they did for other components such as total fat or total calorie consumption (16). As a result, the question still remains as to why sugar-related claims may not always be efficient in promoting healthier food choices.

A potential explanation for these inconsistencies is that the effect of sugar-related labels on consumer choice is mediated by competing subjective consumer perceptions that result from the presence of the label. For example, food labels may elicit various subjective perceptions of food characteristics related to its gustatory properties (sweetness, tastiness), as well as perceived health effects (17, 18), which may not always lead to an increase in WTP (19). However, previous studies on the effects of sugar-related labels have primarily assessed the overall effects of labels on actual purchases, willingness to pay, or purchase intentions for labeled items, without evaluating whether these effects are mediated by subjective perceptions induced by the label. For example, in a systematic review of the sugar-label effects on sugar-sweetened beverage consumption, only 6 of the 21 included studies assessed the healthiness perceptions of the labeled beverages (11), and no study investigated the mediating role of these healthiness perceptions on the relevant outcome measures, such as purchase intentions or the WTP. The scoping review of the front-of-package nutrient warning labels on sugar-sweetened beverages and ultra-processed foods (20) presents similar results: although many studies intended to measure the effects of nutrition labels on consumer behavior, only a few of them focused on the subjective perceptions of healthiness or tastiness induced by the label, and no study directly tested the mediation effects of subjective perceptions on behavioral outcomes.

Furthermore, while a significant body of research has focused on the effects of negatively framed sugar-related warning labels (e.g., “high in sugar”), which primarily function to highlight product’s health risks, less is known about the impact of positively framed content-related claims such as “sugar-free” or “low-sugar”, despite their popularity in many countries (21–23). This divergence is important since these two types of labels may elicit various perceptual mechanisms and inferences about food qualities, resulting in different behavioral outcomes. According to Alcantara et al. (24), positively and negatively framed labels may engage different emotional and cognitive processing mechanisms related to health risk avoidance, with negatively framed labels producing stronger implicit associations with negative health consequences than positively framed labels.

The present study contributes to the existing literature in two significant ways. First, it broadens the understanding of the effects of the sugar-free claims on consumer behavior. Second, it employs the controlled laboratory environment to directly test whether consumers are willing to pay more for products with sugar-free claims than for sugar-containing ones and whether the effects of the sugar-free claims on willingness to pay are mitigated by the opposing influences that such claims may have on the perceived characteristics of the product, such as healthiness, tastiness, and sweetness.

It is well established that various product packaging characteristics provide cues for consumers to make judgments about the healthiness and gustatory characteristics of the items (21, 25, 26). Non-directive product labels, i.e., labels that convey nutritional information without prescribing any action based on this information, constitute an important cue for consumers as well (27). The presence of the sugar-free claim may affect the perception of product characteristics and, correspondingly, the willingness to pay in multiple ways.

First, the sugar-free claim may affect the expectations of how tasty the product is. According to some studies, when participants are presented with a reduced-sugar or other health-related claim, they expect the product to be less tasty (18), less sweet, and less caloric (17). Interestingly, this may be true even for products containing natural sweeteners such as stevia (21). From an evolutionary standpoint, humans and non-human primates learned to associate sweet taste with carbohydrates such as glucose and fructose, as they constitute the primary energy source (28, 29). Therefore, it became essential for survival to associate sugar-enriched food with reward. In both animals and humans, sweet taste has been demonstrated to activate opioid and dopaminergic systems linked to reward processing (30, 31). Sucrose also interacts with neural pathways that serve to transmit the information on the rewarding and nutritional value of food (32). As a result, when a sugar-free label is present on a food product, it might reduce the expectations of how rewarding or energy-rich the food will be. As consumers’ taste perceptions are among the most important determinants of the willingness to pay (33–35), we expected a negative mediation of the sugar-free label effects on willingness to pay through perceived sweetness and tastiness.

Second, the sugar-free claim may raise the expectation of how healthy the product is. Concerns regarding the detrimental effects of excessive sugar consumption have grown globally in recent years. Organizations such as WHO have introduced recommendations on daily consumption of sugar, while governments in many countries have implemented legislations aimed at reducing the consumer sugar intake (36). Although consumers may not always be aware of these recommendations or policies (37), they still seem to link excessive sugar intake to health problems. For example, reported consumers’ associations with sugar included not only sweetness and pleasant taste but also increased body fat, high blood pressure, and diabetes (38). Claims such as “fruit sugar” or “reduced sugar” have been shown to increase perceived product healthiness (39, 40). Existing evidence also suggests that consumers generally are willing to pay more for healthier products. A recent systematic review reported that according to 23 out of 26 included studies, consumers are willing to pay on average 30% more for healthier products (41). Therefore, we expected that perceived healthiness would positively mediate the effects of the sugar-free label on willingness to pay.

Although healthiness and tastiness seem to be generally positively related to the willingness to pay, this relationship is still not always guaranteed. Some studies have reported a negative association between a health-related label and willingness to pay. For example, a recent experiment measuring willingness to pay in a more controlled laboratory setting showed that participants discounted a product when the health label was present, while adding the taste label did not significantly increase their willingness to pay (19). These findings underscore the need for a more thorough investigation of the mechanisms guiding the links between healthiness, tastiness, and the willingness to pay, particularly related to the subjective perceptions of healthiness and tastiness induced by the labels, which is the focus of the present study.

Importantly, perceived healthiness and tastiness may be interrelated due to the “unhealthy-tasty” intuition that some consumers follow, as suggested by previous studies. In particular, consumers may believe that the healthiness and pleasure of product consumption are inversely related (15, 42). They may also perceive a product as less tasty when health information is highlighted on the packaging (21). This may be especially relevant for tasty food products, where it has been demonstrated that labels emphasizing hedonic properties had a greater positive influence on evaluation compared to health labels (43). However, there is a controversy regarding the actual relationship between perceived healthiness and tastiness. Some studies have found that indicating lower sugar content indeed has a positive effect on the product’s perceived healthiness while not affecting its perceived tastiness (44). Other studies have reported a positive association between healthiness and tastiness (8, 45, 46).

Finally, sugar-free labels may give rise to the “halo” effect whereby consumers perceive a product as overall healthy based on a limited amount of nutrient information. For example, in one study vitamin-fortified snacks were perceived as healthier, while participants were less likely to make a healthier snack choice (47). In another study, participants judged a “high-protein” product to contain healthier amounts of other non-protein components (48). It has also been shown that even fabricated meaningless labels like “MUI-free” promote the perception of a product as healthy (49).

In the present study, we test the overall effect of the sugar-free claim on the willingness to pay, as well as the components of this effect mediated by health, taste, and sweetness considerations. One potential confounding factor could be the familiarity with these products. If consumers buy sugar-free products less frequently compared to sugar-containing analogues, they may be less familiar with those products. Studies show that familiarity plays a significant role in purchasing decisions and willingness to pay for a product. For example, familiarity and involvement with a product have been shown to have a positive association with the willingness to pay for organic food (50) and increase willingness to pay for conventional products (51). To avoid the confounding effect of familiarity on willingness to pay, we additionally control for this factor in the study.

In our experiment, participants placed bids in a Becker-deGroot-Marschak (BDM) auction (52), which is a well-established procedure used to derive a valuation of items (19, 53–56). In this auction, participants report the maximum price that they are willing to pay for each product. Next, a random number is generated. If this random number exceeds the participant’s bid, the participant does not receive the product and keeps the money. If this random number is lower than the participant’s bid, the participant buys this product at a price equal to this number. In this auction, the optimal strategy for a participant would be to state the price equal to their true valuation of a product. Offering a price higher than the true value would increase the chances of getting a product while overpaying for it. Offering a price below the true valuation increases the chances of not getting a product at a price a participant would still be willing to pay. Therefore, the auction mechanism is such that it is optimal for a participant to reveal their true valuation of a product. We used real monetary rewards, and one trial was randomly selected at the end of the experiment to be played for real. It was emphasized to the participants that the auction was not hypothetical and would have real consequences for them. Participants were informed that if they won the auction for one randomly selected product, they would be required to pay the price for it and receive that product at the end of the experiment. If they did not win the auction, they would keep the whole endowment received in the beginning of an experiment. Importantly, the stimuli were selected from among the products actually existing on the market to improve the external validity of the study.

Since the usage of the sugar-free label is not legally limited in the country of the experiment, people may have different interpretations of the “sugar-free” labeling. To pre-select the product categories and to assess the most prevalent intuitive connotation that consumers usually associate with the sugar-free label, an online survey on a separate group of participants was conducted prior to the main experiment. The questionnaire study revealed that the majority of participants considered natural sweeteners (like stevia, honey, agave syrup, etc.) to be healthier than refined sugar. The majority of participants also believed that artificial sweeteners were not healthier than refined sugar. As one of the goals of our study was to test the effect of the sugar-free claim on healthiness perception and on willingness to pay mediated by perceived healthiness, we focused specifically on the interpretation of the sugar-free label as indicating the absence of refined sugar in the product but the possible presence of natural sweeteners. However, further research will be needed to study whether the observed effects hold for products containing artificial sweeteners.

Our main hypothesis was that the presence of the sugar-free claim would increase the perceived healthiness of a product, thereby increasing its valuation for a consumer, but would have an opposite effect on the perceived tastiness and sweetness of the product, reducing willingness to pay. Our experimental results support this hypothesis. We conclude that the positive effect of the claim on product valuation is overridden by its negative effect on perceived product sweetness and tastiness, which results in an overall insignificant change in the willingness to pay for labeled products. The mediation analysis revealed that the sugar-free claim had no significant direct effect on tastiness but had a significant indirect negative effect mediated by reduced perceived sweetness. Therefore, we show that while food containing natural sweeteners may be regarded as healthier compared to sugar-containing analogues, lower perceived sweetness and, hence, tastiness reduces the willingness to pay for these products and limits the effectiveness of the sugar-free label in inducing consumer transition to healthier food choices.

## Materials and methods

### Participants

Fifty participants (male = 21, female = 29) between 18 and 51 years (mean age = 26.2, SD = 6.9), completed the experiment. More detailed information on the sample composition by age, gender, education and income can be found in the Supplemental materials. All participants met the standard criteria for participation in the behavioral experiment. The participants had normal or corrected-to-normal vision, did not have any neurological diseases, had no head injuries in the last 5 years, and did not take psychotropic substances. All participants self-reported not having any psychological disorders related to food consumption (for example, an eating disorder) and did not have confirmed diabetes mellitus. Participants were not experts in the field of dietetics (such as doctor, health coach, educator, etc.). Previous research has shown that the more knowledgeable a consumer is about healthy eating habits, the more responsibly they behave when it comes to food choices, steering clear of questionable nutrients, like fast carbohydrates (57). Participants did not follow any diet or food restrictions during the last month and consumed sweet foods (e.g., sweets, chocolate, cookies) in everyday life. All participants were asked not to eat anything 4 h before the experiment to unify their level of hunger (58). The subjects received a reward of 300 monetary units (MU, ~13 USD, based on the BigMac index at the time of data collection) for participating in the experiment and a randomly selected product and an additional reward of up to 150 MU. The conversion rate for MUs to the local currency was 1:1. The participants were recruited via email from the laboratory experiment participation database. The study was conducted at the Centre for Cognition & Decision Making (HSE University). All participants signed the informed consent form before the beginning of the experiment. All procedures were approved by the ethics committee of the HSE University and were performed in accordance with relevant guidelines and regulations.

### Stimuli

Prior to the selection of the experimental stimuli, an online survey (*N* = 100) was conducted aiming to determine the various product groups that people expect to contain the highest amount of sugar and/or sweeteners. In addition, the survey included several questions about the benefits and dangers of natural and artificial sweeteners. The online survey was answered by 100 people (m = 48, *f* = 52) aged 18 to 60 years (mean age = 33.4, SD = 4.1) through the local online survey service. According to the results, the largest amount of refined sugar is thought to be contained in products such as chocolate, cakes, and other confectionery groups (72% of participants) and cookies, crackers, and other bakery groups (46% of participants). In the subsequent study design, various products of these categories were used as stimuli. Participants were also asked to indicate a statement regarding natural sweeteners that they consider correct: (1) Natural sweeteners are healthier than refined sugar; (2) Natural sweeteners are not healthier than refined sugar; and (3) Natural sweeteners are less healthy than refined sugar. The same questions was asked about the artificial sweeteners. 51% of participants believed that natural sweeteners (fructose, honey, etc.) were healthier than refined sugar. 53% of participants believed that artificial sweeteners (xylitol, aspartame, etc.) were not healthier than refined sugar. 48% of participants believed that products labeled “sugar-free” did not contain refined sugar but may contain natural sweeteners (honey, stevia, etc.). Based on these results, the group of products to be used in the experiment was limited to confectionery and bakery products. Sugar-free products were defined as those not containing refined sugar but potentially containing natural sweeteners (stevia, fructose, etc.). Importantly, all products were chosen from those readily available in the local market.

The set of 60 products was selected from the categories defined above. Each of the products displayed costs up to 150 monetary units (MU). Of these 60 products, 30 items contained sugar and 30 items were sugar-free. Each sugar-containing product had a corresponding sugar-free product of a closely similar appearance when presented without packaging (which was the case in the experimental task).

All products were photographed without packaging with a Canon EOS 200D (resolution: 1920 × 1,200 pixels) camera. The packaging was removed since previous studies have found that the presence of packaging, its color, shape, and company label might influence consumers’ behavior (59, 60). Each product was photographed from two different perspectives, and for each product one of the two images was selected in a randomized way. The stimuli were displayed against a white background (resolution: 800 × 600 pixels).

**Figure 1:**
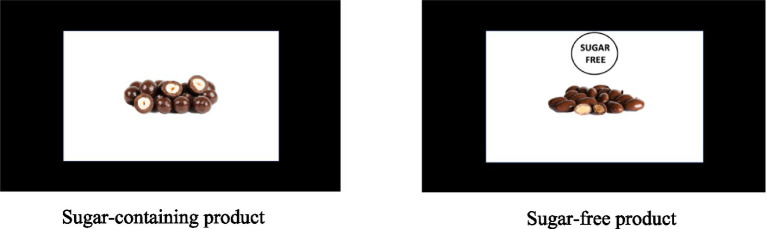
Example of stimuli: sugar-containing product (left), analogous sugar-free product (right).

Sugar-free products were labeled with the sugar-free sign. To avoid the effect of colors on the perception of the stimuli, the label represented a white circle with a black outline and the text “SUGAR FREE” located inside the circle. The label appeared on the screen 1 s after the presentation of a product and remained on the screen until the end of the stimulus presentation. The label was horizontally aligned to be in the center and above the product picture. The position in the center was chosen as the most noticeable for the attention of the participant according to previous studies (61). The stimuli were presented using PsychoPy software (version 3.5). The example of stimuli is shown in Figure 1.

Experimental instructions explained the meaning of the “sugar-free” label as indicating that the product does not contain refined sugar but may contain natural sweeteners (such as honey, fructose, etc.), and that the absence of the label indicated that the product contains refined sugar.

Prior to the experiment, the stimuli were pretested on a separate group of participants (*n* = 12) fully meeting the eligibility criteria for the main experiment to detect whether the two sets of products are perceived similarly when the “sugar-free” label is not shown. The pretest involved the same procedures as the main experiment (described below). The pretest results showed that there was no significant difference in WTP for sugar-free products and their analogous sugar-containing products (mean SF bid = 49.51, mean SC bid = 51.60, *p*-value 0.17). The results also showed that sugar-containing products were perceived as significantly more familiar than their sugar-free analogues (mean for SC = 4.2, mean for SF = 3.7, *p*-value = 0.007). In the other three characteristics, there was no significant difference. Both product groups were almost identical in perceived sweetness (mean for SC = 3.7, mean for SF = 3.8, *p*-value = 0.82). Also, both product groups did not significantly differ in terms of perceived healthiness (mean for SC = 2.1, mean for SF = 2, *p*-value = 0.84) and tastiness (mean for SC = 3.5, mean for SF = 3.4, *p*-value = 0.63).

### Procedure

The experiment consisted of two parts. In the first part, each participant went through the Becker–DeGroot–Marshak (BDM) auction to indicate their WTP for each of the 60 products (52). The products were presented in a fully randomized order to minimize the carryover effects. The presentation order differed for each participant. In the second part, participants were again presented with the same products (in a different random order than in the first part) and had to indicate on a 5-point Likert scale their familiarity with the product (1-not familiar at all, 5-very familiar), its perceived healthiness (1-very unhealthy, 5-very healthy), sweetness (1-not sweet at all, 5-very sweet), and tastiness (1-not tasty at all, 5-very tasty). On each scale, the 5 integer numbers were indicated, and participants had to select the number that best reflects their perception. The order of questions was randomized for each product.

The two parts of the experiment were separated with a break of 10 min in between.

### BDM auction

**Figure 2:**
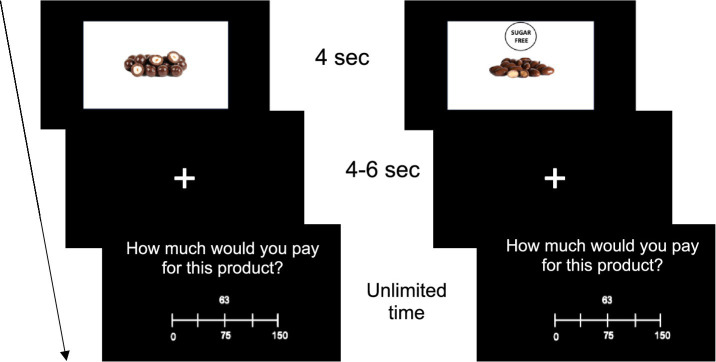
Trial structure for sugar-containing (left) and sugar-free (right) product.

In the BDM auction, each product was shown for 4 s, after which a white fixation cross appeared in the middle of the screen for 4–6 s (randomized across trials). After that, a participant was asked to set a bid for the product. The bid could be selected on the scale from 0 to 150 MU with a step of 1 MU. This scale was selected to ensure that the typical market price for all products would fit within this range. To select a bid, it was necessary to use the mouse cursor on the slider and press the “spacebar” to confirm the selection. Participants were informed that they should set a bid for the quantity of the product shown in the picture. There was no time limit for setting a bid. The trial structure is shown in Figure 2.

At the beginning of the experiment, 150 MU (in addition to the participation fee) were transferred to a participant’s bank account to be used for the purchase of products. This was done to facilitate the perception of this endowment as money already owned by participants, which would promote more thoughtful decision-making during the experiment. Participants were also shown the box containing all the products to make sure they understood that the experimental task was not hypothetical.

Participants were informed that the decisions they would make during the BDM auction would affect the additional reward they would get at the end of the experiment. The following conditions determined this additional reward. At the end of the experiment, one out of 60 products was randomly selected. Then a participant pulled a random capsule with a number from 1 to 150 (step of 1) from the lottery urn. The number in the capsule was considered as the randomly selected price for the product. If the price offered by a participant for this product during the experiment was greater than or equal to the number from the capsule, participants bought the product for the price equal to the number from the capsule by transferring the corresponding amount back to the experimenter. The money that a participant did not spend on the purchase of the product remained in their account. If the price offered by the participant for the product was less than the number from the capsule, they did not receive the product, paid nothing, and kept 150 MU in full on their account. For example, assume the participant indicated a price of 83 MU for the selected product. The participant received the product if the number in the capsule ranged from 0 to 83. The participant did not receive the product if the number fell between 84 and 150. The mechanism of the BDM auction was thoroughly explained to the participants in the instructions. It was also conveyed to them that it was in their best interest to offer a price at which they were actually ready to buy the product. Specifically, participants were explained that offering a price higher than their true valuation would increase their chances of overpayment for a product, while offering a price below their true valuation would lower their chances of getting it at a price that they would be ready to pay. Both of these scenarios would be suboptimal for participants, ensuring that stating their true valuation is incentive compatible.

### Data analysis

#### Statistical tests

We first used a paired *t*-test to test the hypothesis that the average WTP is different between two product categories without controlling for any other product characteristics. The normality of the WTP distributions was confirmed with the Shapiro–Wilk test. Additionally, we used a Wilcoxon signed-rank test to test the hypothesis that products with and without the label differ in the perceived familiarity, sweetness, healthiness, and tastiness. The difference was considered significant when the *p*-value was below 0.05.

#### Mediation analysis

**Figure 3:**
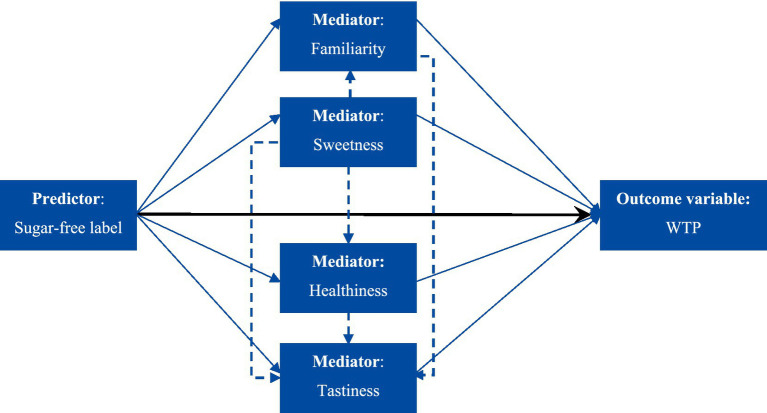
Path diagram describing the structural model for the effects of sugar-free labels on willingness to pay. The black arrow indicates the direct effect of the label on WTP. Solid blue arrows indicate the indirect effects of the label on WTP through each mediator variable. Dashed blue arrows indicate the relationship between mediator variables.

To estimate the sugar-free label’s direct, indirect and total effects on the willingness to pay, we used the structural equation model (SEM). The structural equation modeling technique allows us to take into account the complex nature of interrelations between various characteristics of the stimuli (62). The SEM technique allows us to set up a model where each variable can serve as a dependent variable in some equations and as an explanatory variable in other equations. For example, in our study, perceived sweetness may directly affect willingness to pay, or it may indirectly increase it through increased perceived tastiness. Alternatively, it may decrease perceived healthiness and therefore negatively affect willingness to pay. The complete set of assumed relations between the product characteristics and the WTP is presented in Figure 3.

The structural equation model consisted of five equations. The dependent and explanatory variables for each equation are provided in Table 1. All equations included subject-level and product-level random effects. The product-level random effects were defined as random effects corresponding to each of the 30 pairs of sugar-free and analogous sugar-containing products.

**Table 1:** Description of the structural model including interdependencies between product characteristics and the presence of the label.

Regression number	Dependent variable	Explanatory variables	Control variables	Random effects
(1)	Familiarity	Sugar-free label Sweetness	Gender, Age	Subject-level Product-level
(2)	Sweetness	Sugar-free label	Gender, Age	Subject-level Product-level
(3)	Healthiness	Sugar-free label Familiarity Sweetness	Gender, Age	Subject-level Product-level
(4)	Tastiness	Sugar-free label Familiarity Sweetness Healthiness	Gender, Age	Subject-level Product-level
(5)	WTP	Sugar-free label Familiarity Sweetness Healthiness Tastiness	Gender, Age, Trial number	Subject-level Product-level

Specifically, we expected the sugar-free label to have several indirect effects on the WTP through decreased familiarity with the labeled product, decreased perceived tastiness, decreased perceived sweetness, and increased perceived healthiness of a product. Below we explain each path in greater detail.

The pretest of experimental stimuli revealed that participants are less familiar with the sugar-free products even when the label is not visible. We therefore expected that labeled sugar-free products would be less familiar to participants as well. Previous studies have shown that familiarity with the product is typically positively associated with the willingness to pay for various food categories (50, 51), which underscored the expectation of a negative mediation of the sugar-free label on the WTP through decreased familiarity. The positive association between food familiarity and the WTP could be attributed to the so-called food neophobia, which reduces the acceptance and willingness to pay for novel and unfamiliar foods (63, 64).

Perceived product sweetness and tastiness were expected to be negatively affected by the sugar-free label. The presence of positive sugar-related claims, such as “no sugar” or “low sugar”, was shown to reduce the consumer expectations of how sweet and how tasty the product will be, both for artificial and natural sweeteners (17, 18, 21). As the present study focuses on the food category of sweet snacks, where palatability is one of the major drivers of choice (65, 66), we hypothesized a decrease in perceived tastiness and sweetness, as well as a subsequent decrease in the WTP for labeled products compared to unlabeled ones.

We hypothesized that a sugar-free label would increase the perceived healthiness of the product, which in turn would lead to increased WTP. This effect might occur due to participants realizing negative effects of sugar on their health and thus valuing products with lower amounts of sugar higher, or due to the “health-halo” effect when presenting any positive content-related claim induces the perception of a product as healthier (67, 68).

Finally, we have included a direct effect of the label on the WTP to account for any effects not mediated by the subjective perceptions of sweetness, tastiness or healthiness.

In addition to these main mediation pathways of interest, we hypothesized several direct connections between the mediators.

Since participants might judge the product characteristics, such as sweetness, not only based on the label but also based on the visual cues of the product, perceived sweetness was included as an explanatory variable in the regressions for familiarity, tastiness, and healthiness. If participants are more interested in the hedonic properties of sweet snacks, for which sweetness is one of the major components, they might be more likely to choose snacks that are perceived as more sweet in their daily life, which would increase their familiarity with these products. To take into account this possibility, sweetness was included as an explanatory variable for familiarity. The importance of this link for model fit was also confirmed by the test of directed separation (*p*-value<0.001). Additionally, perceived sweetness was included in the regression for tastiness as a major component of taste in hedonic products like sweet snacks (69) and to the regression for healthiness, as participants might make inferences about healthiness based not only on the label but also on the perceived sweetness.

Familiarity was included as an explanatory variable in the regression for tastiness. Multiple studies have shown that familiar food products are not only preferred more but are often rated higher in tastiness compared to unfamiliar ones (70, 71). Healthiness was included as an explanatory variable for tastiness to directly account for the possibility that participants used the unhealthy-tasty intuition (15, 42). However, since other studies have shown that healthiness may be positively related to tastiness (72), we did not have *a priori* expectations regarding the sign of this relationship in our data.

To estimate this structural equation model, we used the piecewise SEM procedure (73). All calculations were performed in R v4.2.1 software (the packages *piecewiseSEM* and *semEff* were used to estimate equation coefficients, as well as direct, indirect, and total effects, and assess their significance). Fisher’s C statistic was used to test for directed separation and evaluate the goodness-of-fit. Confidence intervals for the direct, indirect, and total effects were obtained using bootstrapping with 1,000 iterations. The effect was considered significant if zero was not included in the bootstrapped confidence interval (CI), with CI defined by the lower (2.5%) and upper (97.5%) boundary obtained by non-parametric bootstrapping.

## Results

### Regression analysis

**Figure 4:**
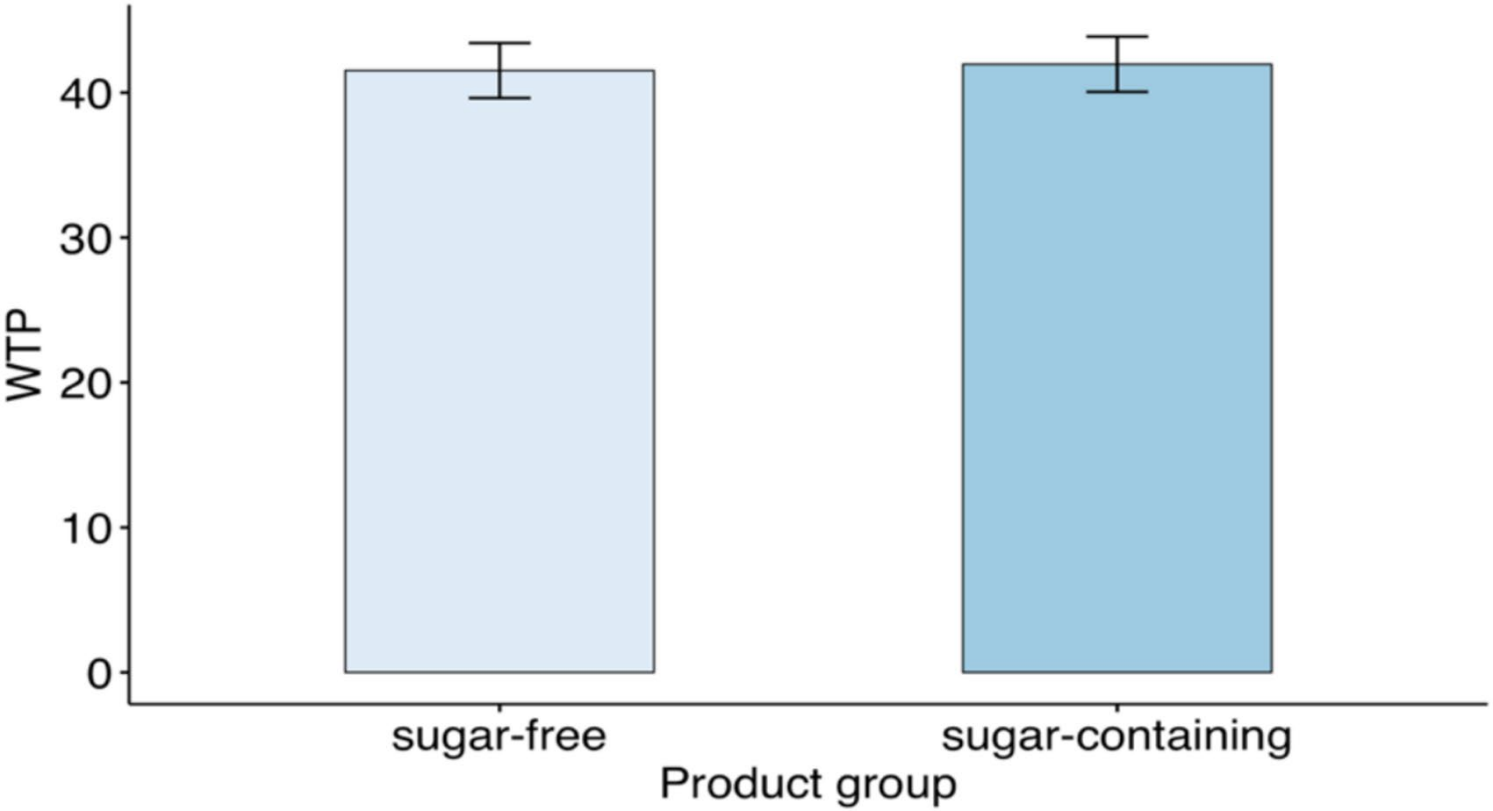
Average WTP for sugar-free products and their analogous sugar-containing products.

First, we analyzed the difference in WTP between sugar-free and sugar-containing products without taking into account any control variables. The average WTP was 41.95 MU for sugar-free products and 41.51 MU for sugar-containing ones (Figure 4). The paired *t*-test did not reveal any significant differences between the two product categories (*p*-value = 0.67).

**Figure 5:**
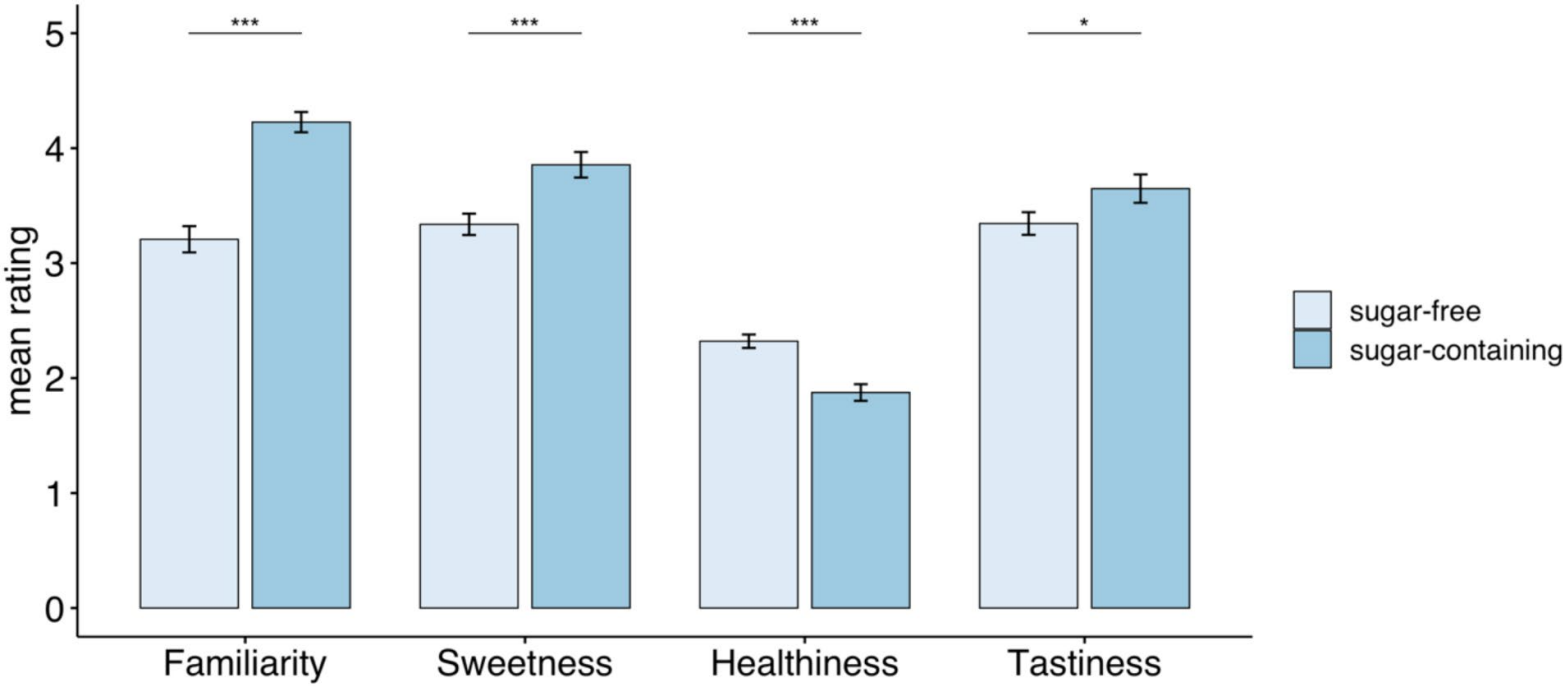
Average ratings of perceived familiarity, sweetness, healthiness and tastiness for sugar-containing and sugar-free products. Whiskers indicate standard error of the mean. *** *p* < 0.001; ** *p* < 0.01; * *p* < 0.05.

The Cronbach’s Alpha computed was 0.91 for product familiarity, 0.94 for perceived sweetness, 0.96 for perceived healthiness, and 0.90 for perceived tastiness, indicating high internal consistency of participants’ responses. We observed significant differences in all four characteristics of products (see Figure 5). Sugar-containing products appeared significantly more familiar to participants compared to sugar-free products (SC: mean = 4.2; SD = 1.3, SF: mean = 3.2; SD = 1.1; *p*-value<0.001). In the presence of the label, the products of the sugar-containing group were rated as significantly sweeter compared to sugar-free products (SC: mean = 3.9; SD = 1.03; SF: mean = 3.3; SD = 1.04; *p*-value<0.001). Sugar-free products received a significantly higher score on healthiness compared to sugar-containing ones (SC: mean = 2.3; SD = 0.8; SF mean = 1.9; SD = 0.9; *p*-value<0.001). The products of the sugar-containing group were rated as tastier compared to sugar-free products (SC: mean = 3.7; SD = 1.2; SF mean = 3.4; SD = 1.2; *p*-value = 0.02).

### Mediation analysis

The piecewise SEM estimation results for each of the five model equations are presented in Table 2.

**Table 2:** Estimation results for structural equation model.

	*Dependent variable*				
	(1) Familiarity	(2) Sweetness	(3) Healthiness	(4) Tastiness	(5) WTP
Constant	3.161^***^	3.678^***^	3.081^***^	1.184^***^	1.445
	(0.256)	(0.241)	(0.271)	(0.255)	(9.658)
Sugar-free label (yes = 1, no = 0)	−0.877^***^	−0.518^***^	0.353^***^	−0.074	2.484^**^
	(0.042)	(0.035)	(0.031)	(0.046)	(0.873)
Familiarity			0.025^*^	0.292^***^	1.274^***^
			(0.013)	(0.018)	(0.364)
Healthiness				0.280^***^	4.352^***^
				(0.026)	(0.515)
Tastiness					5.498^***^
					(0.350)
Sweetness	0.275^***^		−0.230^***^	0.110^***^	1.935^***^
	(0.021)		(0.015)	(0.022)	(0.433)
Trial number					−0.049^*^
					(0.022)
Gender	0.191	0.077	0.099	0.087	7.063
	(0.122)	(0.120)	(0.131)	(0.111)	(4.664)
Age	−0.003	0.006	−0.018	0.009	−0.094
	(0.009)	(0.009)	(0.009)	(0.008)	(0.332)
Observations	3,000	3,000	3,000	3,000	3,000
Log Likelihood	−4,643.879	−4,199.497	−3,546.321	−4,628.289	−13,487.770
Akaike Inf. Crit.	9,301.758	8,410.994	7,108.641	9,274.578	26,997.540
Bayesian Inf. Crit.	9,343.802	8,447.032	7,156.692	9,328.635	27,063.610

*** *p* < 0.001; ** *p* < 0.01; * *p* < 0.05. Standard errors in parentheses.

Fisher’s C statistic indicated that the model fits the data well (*F* = 9,364, *p*-value = 0.313) and there are no missing relationships between variables that should be included to explain the data. As expected, sugar-free products are less familiar to participants and are perceived as less sweet as well as more healthy.

We calculated the total, direct, and indirect effects of the sugar-free label on the willingness to pay with familiarity, sweetness, healthiness, and tastiness as mediators. The results are presented in Table 3.

**Table 3:** Direct, indirect and total effects of the sugar-free label on willingness to pay.

Effect type	Variable	Effect estimate	Std. Err.	Lower CI	Upper CI	*p*-value
Direct	Sugar-free label	2.357	1.018	0.542	4.627	0.021
Indirect	Sugar-free label	−1.838	0.485	−2.815	−0.856	<0.001
Total	Sugar-free label	0.520	1.055	−1.366	2.852	0.622
Mediators	Healthiness	1.281	0.323	0.722	2.018	<0.001
	Sweetness	−0.976	0.278	−1.606	−0.505	<0.001
	Tastiness	−1.298	0.257	−1.985	−0.896	<0.001
	Familiarity	−2.045	0.346	−2.980	−1.492	<0.001

All effects are unstandardized (for standardized effects see Supplemental materials).

The results show that the total effect of the sugar-free label on the willingness to pay is not significant (row 3 of Table 3, bootstrapped CI contains zero). However, this is due to the direct and indirect effects canceling each other out (rows 1 and 2 in Table 3). The presence of the sugar-free label increases the willingness to pay by significantly increasing the perceived healthiness of the product but decreases it via decreased sweetness, tastiness, and familiarity with the product. The significant positive direct effect of the label points at some other factors not included in the model but being important for explaining the overall label effect (see Discussion).

Interestingly, the regression estimation results in Table 2 suggest that there is a positive direct relationship between healthiness and tastiness. However, as sweetness is positively related to tastiness, and sugar-free labeled products are perceived as less sweet, we hypothesized that the negative effect of the label on tastiness is fully mediated by other characteristics such as sweetness and familiarity. The mediation analysis for the effects of the label on tastiness supports this hypothesis (Table 4).

**Table 4:** Direct, indirect and total effect of the sugar-free label on *tastiness*.

Effect type	Variable	Effect estimate	Std. Err.	Lower CI	Upper CI	*p*-value
Direct	Sugar-free label	−0.034	0.037	−0.114	0.035	0.358
Indirect	Sugar-free label	−0.224	0.030	−0.281	−0.162	<0.001
Total	Sugar-free label	−0.258	0.036	−0.333	−0.189	<0.001
Mediators	Healthiness	0.095	0.018	0.064	0.132	<0.001
	Sweetness	−0.072	0.024	−0.127	−0.030	0.003
	Familiarity	−0.271	0.022	−0.314	−0.228	<0.001

All effects are unstandardized (for standardized effects see Supplemental materials).

The mediation analysis for tastiness shows that the sugar-free label does not have a significant direct effect on perceived tastiness. The significant total effect stems from significant indirect effects mediated positively by perceived healthiness and negatively by perceived sweetness and familiarity.

## Discussion

The present study tests the hypothesis that the sugar-free label increases the willingness to pay for a food product. In the present experiment, we employed the Becker-deGroot-Marschak auction procedure to elicit the participants’ willingness to pay in an incentive-compatible way. In this procedure, a participant states their bid for each product, and then a random number is generated to indicate the product price. If a participant’s valuation is higher than the random price, they get the product by paying the price; otherwise, they do not get the product and keep the money. To increase the validity of the results, we used products actually existing on the real market. Additionally, the participants’ choices in the experiment had real monetary consequences for them, as they had an opportunity to either keep the endowment or to buy one of the products depending on their decisions.

The obtained results demonstrate that the sugar-free label increases willingness to pay via significantly increasing the perceived healthiness of the product; yet, the willingness to pay decreases because of lower perceived sweetness and familiarity with the sugar-free product. As these indirect effects act in opposing directions, the total effect of the label on willingness to pay turns out to be insignificant. Interestingly, we find that tastiness is directly positively associated with product healthiness but negatively with sweetness. Hence, this evidence suggests a health-sweetness rather than the health-tastiness tradeoff.

We also observe a direct effect of the label on WTP unexplained by the included mediators. We speculate that there might be several possible sources for this direct effect. First, since we deliberately used products existing on the real market, participants might be familiar with the typical prices of these products seen in supermarkets. Therefore, while selecting a bid, they may rely on the assumption that sugar-free products are usually more expensive than similar sugar-containing products. Although experimental instructions explained in detail how bidding would affect the probability of obtaining the product, the market price could still serve as an unconscious anchor (74).

Second, the awareness about the negative consequences of excessive sugar consumption might have resulted in an unconscious bias against the sugar-containing products, which may not be fully reflected by the indirect effect through perceived (un)healthiness. It has recently been shown that monetary losses may produce both valuation bias (when an option is valued less when the possible loss is higher) and response bias (when an option is rejected simply because it implies a possible loss) (75). A similar decision-making process, although in terms of health rather than monetary losses, may take place in our experimental task. For example, participants may demonstrate a response bias against sugar-containing products, unexplained by health value considerations. This consideration is supported by the recent findings that a narrative about the unhealthiness of refined sugar may successfully decrease the WTP for sugar-containing products without altering the WTP for sugar-free ones (76).

Finally, the presence of the label as a distracting visual stimulus, itself, might have served as a source of bias in valuations of the sugar-free products. For example, previous studies reported that inclusion of an irrelevant but salient stimulus into the stream of outcomes may lead to distorted valuations of these outcomes (77). Various attentional processes may also affect the valuation of a product (78). Further research is needed to clarify the nature of the direct effects of sugar-free labels on product valuations unexplained by perceived product characteristics.

The study adds to the existing literature on the effects of the content-related claims on consumer choices by showing that these claims may not always change the consumers’ choices in the desired direction. These results contrast with some of the previous findings in two major ways. First, the results contradict previously reported findings showing that various nutrition labels may effectively shift consumer preferences towards healthier food options (79). However, it is important to note that many previous studies have considered negatively framed warning labels (such as “high in sugar”) rather than positively framed labels (such as “no sugar” or “low in sugar”) which is the focus of the present study. Many studies focused on the labels providing sugar-related warnings in a graphical form, such as the “traffic-light” system (11). These label format may also be considered as negatively framed as these labels warn about the increased sugar content using the red color. Positively and negatively framed labels may employ very different cognitive mechanisms with negative labels leading to stronger associations with health risks than positively framed ones (24). Many cognitive studies have shown that losses typically loom larger than equally sized gains, and that the avoidance of losses may be a stronger motivator than a possibility of a gain (80). Therefore, it may not be surprising that negatively framed labels turn out to be more effective in changing consumer behavior than positively framed ones.

Second, our results do not support the “unhealthy = tasty” intuition reported in some previous findings (42). Instead, the results of our mediation analysis point at a more nuanced relationship between perceived sweetness, tastiness and healthiness. It is important to note that in our data, perceived tastiness was directly positively related to both healthiness and sweetness, while healthiness and sweetness were related negatively. The mediation analysis for tastiness revealed that the sugar-free label decreases the tastiness judgment by reducing perceived sweetness. Therefore, although tastiness and healthiness may be directly positively associated with each other, the effect of the sugar-free label reduces tastiness via reduced sweetness. Hence, our results suggest that if the “sugar-free” label is to be used on a product it is important to not just present the product as more tasty but to specifically target its perceived sweetness for the label to be effective. The overall effect of the sugar-free label on the perceived product characteristics are in line with the previously reported findings where products with sugar-related claims were rated as healthier, less caloric and less tasty than the non-labeled versions (21).

It should be acknowledged that the insignificant effect of the label on the WTP may have resulted from the low sample size employed in this study, as only 50 participants completed the experiment. The mediation analysis of increasing complexity usually requires higher sample sizes (81). As our study employed a mixed between- and within- study design with each participant providing valuations for each of the 60 products, the total number of data points in the study amounted to 3,000. Although, this is a relatively large number, these observations are not fully independent as they are clustered on a participant level. Therefore, the obtained sample size should still be considered small, which raises concerns regarding the possibility of Type II error. It is possible that the study was underpowered to detect the positive effect of the label on the WTP if this effect is small. Hence, it should not be concluded that the labels are ineffective in shifting consumers’ willingness to pay for the sugar-free products. Rather, additional experiments with a larger sample size are needed to confirm the lack of this effect or to detect its presence.

Several limitations of the study should be mentioned.

A primary limitation of the study is that it was performed in a controlled laboratory environment rather than in real-life conditions. Although the laboratory environment provides an opportunity to directly measure the participants’ preferences and perceptions, in a natural environment, consumer decision-making occurs in a much richer context that our study could not reproduce. To increase the similarity with real-life conditions, we used the incentive-compatible Becker-deGroot-Marschak, procedure where the participants’ choices had real consequences in terms of purchasing a food item at the expense of a part of their monetary endowment. However, many more differences between the laboratory experiment and real-life purchasing conditions are obvious. The channel of purchase, such as whether the item is being sold in a supermarket, or online, or through a HoReCa channel, may have a substantial effect on the overall willingness to pay for the items, as well as on the perception of the “sugar-free” label. For example, the emotional impact of the food consumption may be greatly influenced by the product’s congruency with the context in which it is consumed (82), whereas the presence of others may induce greater consumption of the high-energy foods (83). In a real environment, purchases may be done under time pressure, leading to consumers paying less attention to product information (84). Under time pressure, consumers may be more interested in making impulsive choices of hedonic rather than utilitarian products (85) or may be hesitant to search for sugar-free labeled items located in separate specialized aisles, which would limit the acceptance of such products. Purchasing products through HoReCa channels may increase the desire for products with a high hedonic component, thus decreasing customers’ willingness to pay for “sugar-free” labeled products based on the unhealthy-tasty or unhealthy-sweet intuition. Whether the purchase is made offline or online may also influence attention to nutrition information. Some studies demonstrate that in the online purchases nutrition information promotes healthy food sales (86), while others show that nutrition information does not affect the purchases of tasty carbohydrate-rich food products (87).

Interestingly, recent developments in multisensory perceptions point at the possibility that various visual cues might affect the perception of product sweetness. For example, the color of the plate or the presence of specific colors in the environment increased the perception of sweetness for the sweet products like jelly or cheesecakes (88, 89).

Additionally, in a real-life environment, observing the product brand and packaging may bias the perceptions of healthiness, tastiness and sweetness and therefore change the overall willingness to pay for the product. For example, a recent review of studies on healthy eating points out that food is perceived as less healthy when it comes in glossy packaging or is accompanied by cute designs (90). By contrast, products that appear lighter or prettier are considered healthier (90). Interestingly, some studies show that the brand name alone may not affect the perception of a product as healthy, while a congruent combination of the brand name and product’s shape does (91). Not only the appearance of the food or its packaging may affect perceptions of healthiness or tastiness, but also the appearance of the label (92). We deliberately used colorless labels to explore the effect of information *directly* conveyed by the label without inducing any specific assessment of whether the absence of refined sugar is good or bad. However, colored labels may attract greater consumer attention than black-and-white ones (93), hence shifting the subjective perceptions of product characteristics. For example, green labeling on food may be viewed as signaling healthiness or as a nudge to choose this product over sugar-containing ones (94–96). Interestingly, some visual elements used in sugar-free labels have been shown to affect consumer taste perceptions as well (97). Therefore, the overall effect of the label in this case may depend not only on the meaning of the sugar-free claim but also on the presence of other visual cues. The same concern relates to the fact that, in this study, we used food product photographs without packaging to avoid any biases associated with brand names and logos. However, in real-life circumstances, these product attributes may influence perceptions of healthiness, sweetness, and tastiness in one or another direction and, hence, shift the willingness to pay for the product.

To increase the external validity of the study, all products used in our experiment were selected from among the products that exist in the real market. Sugar-free products were selected to be as similar in appearance to their sugar-containing analogues as possible. However, certain differences in the visual representations persisted and potentially could be biasing the results, since consumers may base their subjective evaluations of sweetness, tastiness, and healthiness based on visual cues. To account for this possibility, we administered a pretest on a separate group of participants where they reported their willingness to pay for each product as well as their subjective perception of the products’ characteristics, without being shown the “sugar-free” label. The results indicated that the participants did not perceive the products as significantly different in any dimension except familiarity, and there was no significant difference in WTP between the product categories on the aggregated level. These results allow us to at least partially confirm that the differences in subjective perceptions observed in the main experiment can be attributed to the label rather than the visual differences between the products. However, due to the small number of participants in the pretest and the main study, it is still possible that visual differences between products played a role in subjective perceptions that were not fully accounted for. If these potential differences were unrelated to the reported subjective perceptions of the product but still played a role in determining the WTP, for example, due to the sugar-free products generally looking more or less attractive than sugar-containing ones, these effects might have been captured in our regression analysis by the direct effect of the label. The direct effect of the label on the WTP was indeed significant and positive, implying that some product characteristics not mediated by the four dimensions measured in the study might have affected participants’ answers.

Although in this experiment we directly asked participants to self-report their familiarity with the products, we did not have any control over participants’ prior experience with the products. In real-life conditions, consumers frequently prefer known and familiar products, which might increase their willingness to pay for them (98). Moreover, studies have shown that when consumers have an opportunity to taste the product, their willingness to pay is defined by their tasting experience rather than by other external cues (99, 100). Since our data show that familiarity with the sugar-free products was systematically lower than with the sugar-containing ones, the participants might have put higher weight on the external cues when evaluating the sugar-free products while putting higher weight on prior experience when evaluating the sugar-containing ones. This unobserved difference might have contributed to the lack of difference in WTP between the product categories. Additionally, not only the product familiarity but also the label familiarity may play a role in subjective evaluations. For example, a recent study found that prior exposure to a regional food label increased the willingness to pay for a food product by 85% (101). In our study, the sugar-free label was artificially created without any reference to the actual market labels. Therefore, our participants were unlikely to have met this particular label in real life, which might have decreased their willingness to pay for the labeled products.

The present study results are limited to specifically confectionary products. This choice was motivated by the fact that this food category is believed to contain the highest amount of sugar, and often the “sugar-free” versions of this food exist on the market. It is therefore important to note that the observed effects of the “sugar-free” label on the WTP and the mediation effects through perceived product characteristics are limited to this specific set of products, despite the fact that in the real market, many other product categories may be labeled as “sugar-free” (e.g., sweet drinks like soda or juices, chewing gum, etc.). As confectionary products may not be generally considered “healthy” due to a high level of non-sugar carbohydrates (e.g., flour), and high amounts of other components like fat, it is possible that the improvement in perceived healthiness caused by the “sugar-free” label was insufficient to induce a significant shift in the WTP. However, this may not be true for other food categories with lower carbohydrate content or for products where healthiness is associated with components other than sugar, such as salt or fat. This might explain the contradiction with some of the previous findings suggesting that consumers are generally willing to pay a premium for products considered to be healthier. Therefore, further research is needed to determine whether a similar effect would be observed in other food categories.

In our study, we used a very narrow definition of the “sugar-free” label, which included only natural sweeteners such as honey, stevia, or agave syrup. Since natural and artificial sweeteners may differ substantially in terms of actual and perceived taste and health benefits, the results of this study cannot be extrapolated to products containing artificial sweeteners. Importantly, artificial sweeteners differ in terms of how closely their taste resembles sucrose (102). Some artificial sweeteners are known to have taste artifacts such as bitterness or chemical flavor, or to be less sweet compared to sucrose, which may be particularly noticeable in products with more complex taste patterns (102). In addition to taste variations, recent studies suggest that artificial sweeteners may involve negative health consequences, especially under long-term consumption, including higher all-cause mortality, type 2 diabetes, hypertension, and cardiovascular disease (103). These differences between natural sweeteners and sucrose may influence subjective consumer perceptions of the sweetness, tastiness, and healthiness of products containing artificial sweeteners, hence biasing their willingness to pay for these products.

Not only the definition of the “sugar-free” label but also the understanding of the label meaning should be appropriate for it to have desirable effects on consumer behavior. Although in our study the instructions for participants clearly indicated the definition of the label, we did not test participants on whether they actually correctly understood or paid proper attention to this information. Therefore, it is still possible that some participants misunderstood the label as indicating artificial sweeteners, which may have introduced bias in the observed effects. In real-life circumstances, nutrition knowledge variations between consumers may lead them to either ignore the label information (104) or dismiss the labeled products due to insufficient information on the label (19). In our experiment, we selected participants who had no nutrition-related education and did not intentionally limit their sugar consumption in their daily lives, but we did not test how much knowledge they generally had about the consequences of excessive sugar consumption or how much attention they pay to the sugar content of food during daily purchases. Our results, therefore, do not provide insights about how these consumer characteristics would affect the mediation effects of the product perceptions on the willingness to pay.

As sweet snacks constitute a group of products that are high in calories, and sugar is a major source of energy in food, current energy needs may drive participants’ choice in favor of higher-calorie food (105) and their willingness to pay for these products. In our study, we asked participants to fast for 4 h prior to the experiment to minimize differences in the level of hunger; however, the actual fasting time was not controlled for, which might have introduced distortions in the willingness to pay for sugar-containing versus sugar-free products. For example, participants with higher hunger levels may have increased perception of the sweetness or tastiness of the sugar-containing products or may have discounted the health-related benefits of sugar-free products, while the opposite may be true for less hungry participants. It is also known that being hungry might increase the tendency to behave impulsively in the purchasing context, leading to increased willingness to pay for less healthy products (106, 107).

Finally, as our sample was small and culturally homogenous, the extrapolation of these results to other countries and cultures should be done with caution. Cultural differences may play a considerable role in the label’s effect on willingness to pay. For example, one study has shown that cultural differences in food sustainability concerns led to differences in attitudes to food labeled as sustainable between European, Asian, and US consumers (108). Wine consumption habits have been shown to not only influence the WTP for wine but also moderate the effect of label information on the WTP (109–111). Consumption habits in general, and understanding of the consequences of excessive sugar in particular, may vary between cultures (112, 113). It is important to note that Russia is a country with higher-than-average consumption of sugar compared to other countries across the world, with annual per capita consumption reaching 35–40 kg and remaining relatively stable over the past several years (114, 115), despite efforts on the federal level to increase the acceptance of healthier eating habits. According to some reports, more than 25% of adolescents and adults consume sugary carbonated drinks or more than 10 tablespoons of sugar per day (116). The level of health literacy about diet remains relatively low: while more than half of the consumers report that they follow the principles of a healthy diet, 20% of those believing their diet is healthy consume fast food on a regular basis (117). Moreover, unlike in many other countries (118), the local regulatory policy does not establish any official restrictions on the use of sugar-free labels, which means that there is no one well-defined understanding of the label as well as no single established label design that consumers would be accustomed to. As a result, in a real market, many consumers may have low understanding and trust in such labels, which would reduce their willingness to pay for labeled products. Therefore, further studies are needed to explore whether similar patterns will be observed in other Western and Eastern cultures.

The present study provides evidence that the sugar-free labeling may not be effective in increasing the willingness to pay for sugar-free products due to the opposing effects that the sugar-free claim may induce on perceived product characteristics such as healthiness, tastiness, and sweetness. Although sugar-free products might indeed be perceived as healthier, they are at the same time expected to be less sweet and less tasty, which might decrease willingness to pay and hence limit the effectiveness of the sugar-free claims in changing consumer choices towards healthier food options. Reduced familiarity with sugar-free products contributes to this effect. From a practical standpoint, the study results suggest that introducing a sugar-free claim on a food package may not be sufficient to induce a switch of consumer choices from sugar-containing to sugar-free analogues, and additional measures increasing sweetness perceptions might be needed.

Further studies may explore various ways in which the perceived sweetness or tastiness of the sugar-free products may be increased by using multisensory design cues. As mentioned earlier, various packaging characteristics, such as the shape, color, or surface appearance, as well as the visual characteristics of the label, may shift the perception of the product’s healthiness. Focusing on a multisensory approach to label or packaging design may provide additional benefits since it has been shown that a combination of different sensory cues may have a greater effect on perception and subsequent choices than each cue in isolation (119, 120), though a lack of evidence for multisensory integration was observed in some contexts as well (121). Furthermore, cultural differences in label perception may be linked to differences in the amount of trust that consumers have in label information. Differences in the level of regulation applied to the sugar-content-related labels may fuel skepticism towards such labels in some countries, therefore reducing the label’s effectiveness in promoting healthier diets. Further studies are needed to explore the extent to which a lack of trust in label information would influence the willingness to pay for labeled products.

## Data Availability

The datasets presented in this study can be found in online repositories. The names of the repository/repositories and accession number(s) can be found at: https://github.com/openaccessdata/sugar_free_WTP.

## Author’s note

This manuscript has been released as a preprint at arXiv: https://doi.org/10.48550/arXiv.2311.09885.
