# Supplementary material for: Opposing effects of sugar-free claims on perceived healthiness and sweetness reduce consumers’ willingness to pay for sugar-free products
Source: Front Nutr. 2025 Oct 31;12:1644753. doi: 10.3389/fnut.2025.1644753 (PMC12616639; doi:10.3389/fnut.2025.1644753)
Supplement: Supplementary file 1 [file Table_1.docx]

SUPPLEMENTAL MATERIAL

| **Age** | | **18-29** | **30-39** | **>40** | **Total** |
| --- | --- | --- | --- | --- | --- |
| Male | # | 15 | 5 | 1 | 21 |
|  | % | 71.4% | 23.8% | 5% | 100% |
| Female | # | 23 | 4 | 2 | 29 |
|  | % | 79.3% | 13.8% | 6.9% | 100% |
| Total | # | 38 | 9 | 3 | 50 |
|  | % | 76% | 18% | 6% | 100% |

**Table S1.** Sample composition by age and gender.

| **Education level** | **Number of participants** | **Percentage** |
| --- | --- | --- |
| Finished primary school | 0 | 0% |
| Finished secondary school | 0 | 0% |
| Undergraduate program student | 16 | 32% |
| Master program student | 9 | 18% |
| Completed bachelor degree or equivalent | 0 | 0% |
| Completed master degree or equivalent | 25 | 50% |

**Table S2.** Sample composition by education.

| **Income range** | **Number of participants** | **Percentage** |
| --- | --- | --- |
| Less than 500 | 4 | 14% |
| 500 - 1700 | 9 | 31% |
| 1700 - 3500 | 10 | 34% |
| 3500 - 5000 | 4 | 14% |
| More than 5000 | 2 | 7% |

**Table S3.** Sample composition by affordable monthly income (only 29 participants provided this information). Currency units are in USD converted from the local currency by the purchasing power parity.

|  | | | | | | | |
| --- | --- | --- | --- | --- | --- | --- | --- |
|  | Effect type | Variable | Effect estimate | Std. Err. | Lower CI | Upper CI | P-value |
|  | | | | | | | |
|  | DIRECT |  |  |  |  |  |  |
|  |  | Sugar-free label | 0.042 | 0.018 | 0.010 | 0.080 | 0.02 |
|  | INDIRECT |  |  |  |  |  |  |
|  |  | Sugar-free label | -0.033 | 0.009 | -0.049 | -0.015 | < 0.001 |
|  | TOTAL |  |  |  |  |  |  |
|  |  | Sugar-free label | 0.009 | 0.019 | -0.025 | 0.048 | 0.636 |
|  | MEDIATORS |  |  |  |  |  |  |
|  |  | Healthiness | 0.023 | 0.006 | 0.013 | 0.035 | < 0.001 |
|  |  | Sweetness | -0.017 | 0.005 | -0.029 | -0.009 | < 0.001 |
|  |  | Tastiness | -0.023 | 0.005 | -0.035 | -0.016 | < 0.001 |
|  |  | Familiarity | -0.036 | 0.006 | -0.050 | -0.027 | < 0.001 |
|  | | | | | | | |

**Table S4.** Direct, indirect and total effect of the sugar-free label on willingness to pay (standardized). The “Mediators” section presents the components of the total effect mediated by each of the four product characteristics. Lower and Upper CIs correspond to the lower (2.5%) and upper (97.5%) confidence interval boundaries obtained by non-parametric bootstrapping.

|  | | | | | | | |
| --- | --- | --- | --- | --- | --- | --- | --- |
|  | Effect type | Variable | Effect estimate | Std. Err. | Lower CI | Upper CI | P-value |
|  | | | | | | | |
|  | DIRECT |  |  |  |  |  |  |
|  |  | Sugar-free label | -0.014 | 0.014 | -0.044 | 0.014 | 0.317 |
|  | INDIRECT |  |  |  |  |  |  |
|  |  | Sugar-free label | -0.089 | 0.012 | -0.112 | -0.063 | < 0.001 |
|  | TOTAL |  |  |  |  |  |  |
|  |  | Sugar-free label | -0.103 | 0.014 | -0.131 | -0.075 | < 0.001 |
|  | MEDIATORS |  |  |  |  |  |  |
|  |  | Healthiness | 0.038 | 0.007 | 0.026 | 0.052 | < 0.001 |
|  |  | Sweetness | -0.029 | 0.010 | -0.051 | -0.012 | 0.004 |
|  |  | Familiarity | -0.108 | 0.009 | -0.125 | -0.090 | < 0.001 |
|  | | | | | | | |

**Table S5.** Direct, indirect and total effect of the sugar-free label on *Tastiness* (standardized). The “Mediators” section presents the components of the total effect mediated by each of the other product characteristics. Lower and Upper CIs correspond to the lower (2.5%) and upper (97.5%) confidence interval boundaries obtained by non-parametric bootstrapping.
